# Coding-Complete Genome Sequences of Six Influenza Type A Strains Circulating in Lithuania in the 2009–2010 Epidemic Season

**DOI:** 10.1128/MRA.01274-20

**Published:** 2021-01-07

**Authors:** Lukasz Rabalski, Boguslaw Szewczyk, Kęstutis Zagminas, Algirdas Griskevicius, Krzysztof Lepek

**Affiliations:** a Laboratory of Recombinant Vaccines, Intercollegiate Faculty of Biotechnology, University of Gdansk and Medical University of Gdansk, Gdansk, Poland; b Vilnius University, Faculty of Medicine, Public Health Institute, Vilnius, Lithuania; c National Public Health Surveillance Laboratory, Vilnius, Lithuania; Queens College

## Abstract

Here, we report the coding-complete genome sequences of six influenza A(H1N1) strains that were detected in Vilnius, Lithuania, among patients exhibiting influenza-like symptoms during the 2009–2010 epidemic season, within national influenza surveillance. Several mutations were found in genes encoding hemagglutinin and neuraminidase, in comparison with the A/California/07/2009 reference strain (GenBank accession numbers NC_026433 and NC_026434).

## Abstract (precis)

Here, we report the coding-complete genome sequences of six influenza A(H1N1) strains that were detected in Vilnius, Lithuania, among patients exhibiting influenza-like symptoms during the 2009–2010 epidemic season, in a national influenza surveillance. Several mutations were found in genes encoding hemagglutinin and neuraminidase, in comparison with the A/California/07/2009 reference strain (GenBank accession numbers NC026433 and NC026434).

## ANNOUNCEMENT

Belonging to the family *Orthomyxoviridae*, genus *Alphainfluenzavirus*, the influenza type A virus causes annual human and animal influenza. The manifestation of the disease may take the form of seasonal waves of cases, local epidemics, or global pandemics caused by the emergence of antigenically different strains (1).

Lack of immunity to such strains in the human population is caused by continuous genetic variation of the virus, which is based on phenomena such as reassortment (exchange of genetic segments between two different viruses), antigenic shift (changes of genetic segments encoding the major surface antigens), and antigenic drift (slow accumulation of small variations in sequences encoding the major surface antigens) (2, 3). Therefore, it is important to not only keep tracking the current genetic diversity of these viruses but also take a look at changes that occurred in the past to have a full set of data for accurate estimation of the antigenic drift and what course it may take (4, 5).

Viral RNA was isolated, using the RNeasy purification kit (Qiagen), from oropharyngeal swabs collected by the National Public Health Surveillance Laboratory of Lithuania during the 2009–2010 epidemic season from patients showing influenza symptoms such as fever, coughing, headache, and chills. The Vilnius Regional Bioethics Committee approved the study protocol; this was deemed minimal risk, and the requirement for written consent was waived. The samples were positively diagnosed as A(H1N1) with a reverse transcription-quantitative PCR (RT-qPCR) assay using primers and probes recommended by the World Health Organization (6, 7). For all A(H1N1) genomic segments, reverse transcription and PCR amplification were performed using PathAmp FluA reagents (8). Postreaction cleanup was done on Agencourt AMPure XP magnetic beads (Beckman Coulter). We performed full genome sequencing on the MiSeq platform (Illumina) with v3 sequencing chemistry. For genomic library preparation, a Nextera XT DNA sample preparation kit (Illumina) was used. Paired-end reads of the target size 2 × 300 bp were generated (isolate 1167, 2,514,242 reads; isolate 1172, 1,185,476 reads; isolate 1241, 1,758,300 reads; isolate 1305, 1,675,062 reads; isolate 1319, 2,687,104 reads; isolate 1324, 1,299,004 reads). Data were gathered as fastq files and further analyzed with Geneious Prime v20.2 software (Biomatters) using integrated tools (trimming, i.e., deleting 3′ and 5′ ends with an error probability limit of 0.05) and the BBNorm package v38.84 (https://sourceforge.net/projects/bbmap) (error correction and normalization, i.e., correcting substitution errors based on quality, discarding reads that have a probable coverage of 2× or less, and reducing the number of reads that have a probable coverage of 40× or more). *De novo* assembly of contigs was performed using a medium sensitivity script. Final contigs were trimmed to the size of the reference genome (GenBank accession numbers are provided in the footnote of Table 1).

**TABLE 1:** Accession numbers and characteristics of influenza A(H1N1) isolates deposited in GenBank

Isolate	Segment	Segment size (bp)	Similarity to reference (%)^*a*^	G+C content (%)	Accession no.
1167	PB2	2,280	99.65	44.6	MW185783
	PB1	2,274	99.91	42.0	MW185784
	PA	2,151	99.70	44.1	MW185785
	HA	1,701	99.53	40.6	MW185786
	NP	1,497	99.70	46.0	MW185787
	NA	1,410	99.65	42.1	MW185788
	M	982	99.69	47.1	MW185789
	NS	863	99.65	43.7	MW185790
1172	PB2	2,280	99.74	44.7	MW185791
	PB1	2,274	99.96	42.0	MW185792
	PA	2,151	99.56	44.1	MW185793
	HA	1,701	99.59	40.7	MW185794
	NP	1,497	99.43	45.8	MW185795
	NA	1,410	99.57	42.0	MW185796
	M	982	99.69	47.0	MW185797
	NS	863	99.54	43.8	MW185798
1241	PB2	2,280	99.65	44.6	MW185828
	PB1	2,274	99.60	42.2	MW185829
	PA	2,151	99.74	44.2	MW185830
	HA	1,701	99.59	40.7	MW185831
	NP	1,497	99.30	45.9	MW185832
	NA	1,410	99.65	42.1	MW185833
	M	982	99.80	46.9	MW185834
	NS	863	99.77	43.8	MW185835
1305	PB2	2,280	99.61	44.7	MW185803
	PB1	2,274	99.60	42.3	MW185806
	PA	2,151	99.70	44.1	MW185804
	HA	1,701	99.41	40.5	MW185800
	NP	1,497	99.37	46.0	MW185805
	NA	1,410	99.65	42.1	MW185799
	M	982	99.80	46.9	MW185802
	NS	863	99.88	43.7	MW185801
1319	PB2	2,280	99.65	44.7	MW185811
	PB1	2,274	99.60	42.2	MW185814
	PA	2,151	99.74	44.2	MW185812
	HA	1,701	99.47	40.6	MW185808
	NP	1,497	99.37	46.0	MW185813
	NA	1,410	99.65	42.1	MW185807
	M	982	99.80	46.9	MW185810
	NS	863	99.88	43.7	MW185809
1324	PB2	2,280	99.65	44.6	MW185819
	PB1	2,274	99.60	42.2	MW185822
	PA	2,151	99.74	44.2	MW185820
	HA	1,701	99.47	40.6	MW185816
	NP	1,497	99.37	46.0	MW185821
	NA	1,410	99.65	42.1	MW185815
	M	982	99.80	46.9	MW185818
	NS	863	99.88	43.7	MW185817

a Similarity to reference strain A/California/07/2009 (M segment, GenBank accession number NC_026431; NS segment, NC_026432; HA segment, NC_026433; NA segment, NC_026434; PB1 segment, NC_026435; NP segment, NC_026436; PA segment, NC_026437; PB2 segment, NC_026438).

All sequences were aligned, using MAFFT v7.388, against the A/California/07/2009 reference strain circulating among people at that time (9–12). We observed a high degree of similarity (>99%) among all six strains as well as the reference strain (similarity levels are presented in Table 1). These analyses revealed several single-nucleotide polymorphisms that alter the amino acid sequences in two major surface glycoproteins, namely, hemagglutinin (HA) and neuraminidase (NA), i.e., V24I (isolate 1305), R45K (isolates 1319 and 1324), V47G (isolates 1319 and 1324), S74N (isolate 1167), P83S (all isolates), S203T (all isolates), A215V (isolate 1305), I321V (isolates 1167, 1172, 1241, and 1305), I321F (isolates 1319 and 1324), and E374K (all isolates except isolate 1167) in HA and G41V (isolate 1172), I46T (isolate 1167), V106I (all isolates), and N248D (all isolates) in NA. The S74N, P83S, S203T, and A215V substitutions are localized in epitope regions of HA or the immediate vicinity (13). The V47G and S74N substitutions were characterized earlier in our findings, in A(H1N1) isolates collected in Taiwan in 2010 to 2011 (14). None of the isolates harbored the NA drug resistance mutations in the NA gene (15).

### Data availability.

The coding-complete genome sequences of influenza A(H1N1) strains isolated in Vilnius, Lithuania, in 2009 were deposited in GenBank under the accession numbers listed in Table 1. The raw sequencing reads were deposited in the NCBI Sequence Read Archive (SRA) under the accession numbers SRR13000260, SRR13000261, SRR13000262, SRR13000263, SRR13000264, and SRR13000265 and the BioProject accession number PRJNA675103.
